# A Study on the Correlation Between Endoscopic Findings and Symptoms of Gastroesophageal Reflux Disease (GERD)

**DOI:** 10.7759/cureus.24361

**Published:** 2022-04-21

**Authors:** Rajesh Domakunti, Yeshwant R Lamture

**Affiliations:** 1 General Surgery, Jawaharlal Nehru Medical College, Wardha, IND

**Keywords:** reflux esophagitis, heartburn, regurgitation, epigastric pain, non-erosive reflux disease, obesity

## Abstract

Background

Gastroesophageal reflux disease is a common disorder affecting a large section of the community. In addition, the numerous complications of chronic gastroesophageal reflux disease (GERD) necessitate adequate diagnosis and treatment of this common entity. Thus, to analyze the spectrum of GERD on the basis of symptoms and endoscopic findings along with the contributory effects of various risk factors including obesity, this specific study has been carried out.

Study design

A descriptive type of observational study was conducted on the patients visiting the outpatient department (OPD) at Acharya Vinoba Bhave Rural Hospital (AVBRH), affiliated with Datta Meghe Institute of Medical Sciences (DMIMS), Wardha, Maharashtra. The clinical symptoms, suspected risk factors, and endoscopic findings of the patients were studied, assessed, and correlated.

Methods and material

Patients, more than 18 years of age complaining of a minimum of two typical symptoms of GERD for at least one month, were included in the study. Demographic data, clinical symptomatology, and personal history of the patients were noted. All the patients were subjected to esophagogastroduodenoscopy, and findings were recorded. Correlation and analysis were done on clinical and endoscopic findings.

Statistical analysis

This descriptive study has been conducted after the approval of the Ethics Committee Department of Medical Education, Jawaharlal Nehru Medical College, Deemed University, Sawangi (DMIMS(DU)/IEC/Sept-2019/8403). The outcomes were recorded and analyzed at the end of the study using a Microsoft Excel Spreadsheet (version 16.40, Microsoft Corporation, Redmond, Washington, USA).

Results and conclusion

A total of 100 patients were included in the study. A number of male patients (58%) were more than that of female patients. Most of the patients were in the age group of 30-60 years (70%). The most common symptoms were epigastric pain (78%), regurgitation (71%), and heartburn (63%). Forty-five percent of the patients had erosive lesions suggestive of reflux esophagitis on endoscopic evaluation.

## Introduction

Primary health care practitioners come across various common diseases during their medical service. One such common disorder is gastroesophageal reflux disease (GERD) [1]. It is more common in the Western world with a prevalence of around 10%-20%, while in Asia, it is 5% lesser compared to the Western countries [2]. In India, the prevalence of GERD is 7.6% as per the multicentric study done by Bhatia et al. in 2011 [3]. However, in the past 10 years, there is an increasing trend in the prevalence of GERD in Asian countries including India. Improved socioeconomic status of the population along with the adoption of Western culture resulting in lifestyle modifications could be the probable cause for it [4].

GERD is a disease constituting symptoms or complications developed due to reflux of stomach contents back into the esophagus [5]. This disease entity is thought to be much more common than what is registered in various studies and literature as many of the patients suffering from the disease do not seek medical advice. Instead, they medicate themselves without doctors' prescription with over-the-counter drugs from pharmacies to get relief from symptoms. Despite this, 85% of the patients who have approached medical advice are experiencing symptoms of one year and more, while 59% have the duration of symptoms of five years or more [1]. People seeking medical advice are likely to constitute only the tip of the iceberg of disease. Henceforth, the natural history of the disease is still not unambiguous [6]. In the era of modern science and technology with advanced modalities in the field of diagnostic centers and laboratories, still, appropriate history-taking plays a pivotal role in the approach to the diagnosis of GERD [7].

Characteristic symptoms commonly found and suggestive of GERD are acid regurgitation and heartburn [6]. However, even epigastric pain is also considered an important clinical feature. A usual diagnosis for GERD is made on the basis of classical symptomatic history with a probability of a positive response to medical therapy by antacids or antisecretory agents [5]. GERD constitutes a wide spectrum of diseases. Its manifestations are due to mucosal layer abnormality consisting of infiltration of squamous epithelium with inflammatory cells, basal layer thickening, and sloughing of surface epithelial cells. This process of inflammation may further progress to cause erosive esophagitis and complications like hemorrhage or stricture formation. Metaplastic columnar epithelium (Barrett’s esophagus) is also a complicated form of GERD which carries a higher risk for malignancy (adenocarcinoma) by 30-40 times in patients than in the general population [8].

In an uncomplicated GERD with classical clinical history on evaluation of the patient, prophylactic medical treatment with antacids/antisecretory drugs can be tried. If the patient does not respond to empirical therapy or clinical evaluation reveals features suggesting complicated GERD or at high risk for developing esophageal adenocarcinoma, detailed evaluation with upper gastrointestinal (GI) endoscopy, ambulatory pH monitoring, esophageal manometry, and impedance testing should be considered [5]. Due to the limited evidence, the age-old traditional concept of considering GERD existence as a spectrum of diseases is being challenged recently. It has been claimed that this concept has hindered the progress in understanding the pathophysiology of the symptoms and disease. A new concept has been framed, wherein the population with GERD is divided into three distinct subgroups: non-erosive reflux disease (NERD), erosive reflux disease (ERD), and Barrett’s esophagus (BE). By categorizing in this way, the focus of management is completely shifted from esophageal mucosal injury to symptoms of GERD [9]. This type of morphological diagnosis has led endoscopy to become a major weapon to evaluate the GERD and its consequences, specifically in population-based mass screening [10]. Furthermore, for patients with chronic classic symptoms suggesting GERD, endoscopic evaluation for Barrett’s esophagus as well as adenocarcinoma of the esophagus has been recommended nowadays [11]. Reversely, the presence of erosive changes in the esophagus on endoscopy due to reflux disease highlights the patient regarding the risk of chronicity of the disease [12].

Even after various research, the literature is still confusing in regards to the correlation between endoscopic findings and clinical symptoms of GERD. Especially, there are few studies in Indian literature regarding this. Present India is facing a problem of obesity/overweight rather than undernutrition/malnutrition. Obesity itself being a disease entity has now become one of the important risk factors for GERD. Establishing a strong association between GERD and body mass index (BMI) which is one of the best indicators of obesity is very much important. Thus, to analyze the spectrum of GERD on the basis of symptoms and endoscopic findings along with the contributory effects of various risk factors including obesity, this specific study has been carried out.

## Materials and methods

Inclusion criteria

Inclusion criteria are as follows: 1. patients willing to provide informed consent before participation; 2. patients presenting to general surgery/gastroenterology outpatient department (OPD) consistent with symptoms (typical/atypical) suggestive of GERD: a. typical symptoms: heartburn, regurgitation, and epigastric pain, and b. atypical symptoms: hoarseness, sore throat, bloating, belching, dysphagia, and vomiting; 3. patients >18 years of age; 4. patients with at least two symptoms for more than one month; and 5. patients able to attend for diagnostic endoscopy within seven days of consultation.

Exclusion criteria

Exclusion criteria are as follows: 1. previously diagnosed GERD patients who are on treatment; 2. pregnancy; 3. participation in a clinical study within the previous month; 4. previous gastric or upper gastrointestinal surgery; 5. patients with other serious conditions including Zollinger-Ellison syndrome and inflammatory bowel disease; 6. patients who had other significant systemic illnesses including coagulopathy; and 7. diagnosed case of upper GI malignancy.

A patient with symptoms suggestive of GERD was thoroughly evaluated by complete general physical examination, systemic examination, and upper GI endoscopy. Clinical symptoms were assessed as per the presence of duration, frequency, severity, and type of symptoms. Upper GI endoscopy procedure includes keeping the patient Nil by Mouth (NBM) for six hours prior to the procedure, application of local anesthetic spray at the posterior pharyngeal wall, placement of mouthpiece, making a patient lie on his/her left side, and inserting the endoscope into mouth. This procedure takes around 15-30 minutes following which the patient will be kept under observation for about an hour.

Endoscopic findings were noted which can be as follows: 1. esophageal erosions which will be graded as per the Los Angeles (LA) classification system as follows: Grade A: more than one mucosal break no longer than 5 mm, none of which extends between the tops of the mucosal folds; Grade B: more than one mucosal break more than 5 mm long, none of which extends between the tops of two mucosal folds; Grade C: mucosal breaks that extend between the tops of two or more mucosal folds, but involve less than 75% of esophageal circumference; and Grade D: mucosal breaks involving at least 75% of the esophageal circumference; and 2. other abnormal findings like Barrett’s esophagus, hiatus hernia, carcinoma (CA) esophagus, and stricture esophagus. These endoscopic findings were correlated with symptoms of GERD.

Statistical analysis

This descriptive study was conducted after the approval of the Ethics Committee Department of Medical Education, Jawaharlal Nehru Medical College, Deemed University, Sawangi (DMIMS(DU)/IEC/Sept-2019/8403). This descriptive study was conducted after obtaining written consent from the patient. The outcomes were recorded and analyzed at the end of the study using a Microsoft Excel Spreadsheet (version 16.40, Microsoft Corporation, Redmond, Washington, USA).

## Results

A total of 100 cases were enrolled for this study from the Departments of General Surgery, General Medicine, and Gastroenterology at Acharya Vinoba Bhave Rural Hospital (AVBRH), affiliated with Jawaharlal Nehru Medical College (JNMC), Sawangi (Meghe), Wardha, Maharashtra.

Gender distribution

In our study, out of 100 cases, 58 patients were male, while 42 were female (Table 1).

**Table 1:** Gender distribution of study subjects

Gender	Frequency	Percentage
Male	58	58
Female	42	42
Total	100	100
c^2^ value	2.56	
p-value	0.10 (non-significant)	

Age distribution

In our study, the age of the patient varied between 18 and 80 years. Most of the cases belonged to the age group of 30-60 years (70%) with a mean age and standard deviation of 44.82 and 14.57, respectively (Table 2).

**Table 2:** Age distribution of study objects

Age Group	Frequency	Percentage
<30 years	16	16
30-60 years	70	70
>60 years	14	14
Total	100	100
Mean±SD	44.82±14.57 (18-80 years)	
c^2^ value	60.56605761	
p-value	7.05095E-14 (significant)	

Symptomatology​​​

In our study, out of 100 cases, 63 patients presented with chief complaints of heartburn, 71 patients with regurgitation, and 73 patients with epigastric pain. Belching was found in 16 patients, being the most common among atypical symptoms (Table 3).

**Table 3:** Clinical spectrum of GERD in study subjects GERD: gastroesophageal reflux disease.

Symptoms	Frequency	Percentage
Typical		
Heartburn	63	63
Regurgitation	71	71
Epigastric pain	78	78
Atypical		
Dysphagia	9	9
Hoarseness	8	8
Sore throat	5	5
Bloating	8	8
Belching	16	16
Vomiting	7	7

Study subjects were further categorized based on their duration, frequency, and severity of symptoms. Duration of symptoms between four and six months was found in 52 cases, while less than four months and more than six months were found in 32 and 16 cases, respectively (Table 4). Daily symptoms were present in 31 cases, while several times in a week, once in a week, and once in a month symptoms were found in 28, 24, and 17 cases, respectively (Table 5). Out of 100 cases, 59 patients had a mild severity in their symptoms, while moderate and severe symptoms were seen in 24 and 17 patients, respectively (Table 6).

**Table 4:** Distribution of patients based on duration of symptoms

Duration of Symptom	Frequency	Percentage
Less than four months	32	32
Four to six months	52	52
More than six months	16	16
Total	100	100

**Table 5:** Distribution of patients based on frequency of symptoms

Frequency of Symptom	Frequency	Percentage
Once in a month	17	17
Once in a week	24	24
Several times in a week	28	28
Daily	31	31
Total	100	100

**Table 6:** Distribution of patients based on severity of symptoms

Severity of Symptom	Frequency	Percentage
Mild	59	59
Moderate	24	24
Severe	17	17
Total	100	100

Personal history

Out of 100 cases in our study, 10 patients had a sedentary lifestyle and eight patients had a history of insomnia. Among addictive history, the percentage of cases with alcohol consumption, smoking, and tobacco consumption was 21%, 15%, and 14%, respectively (Table 7).

**Table 7:** Patients with personal history

Personal History	Frequency	Percentage
Sedentary lifestyle	10	10
Insomnia	8	8
Addictions		
Alcohol	21	21
Smoking	15	15
Tobacco	14	14

Nutritional status

Body mass index (BMI) was used to measure the nutritional status of the cases taken in our study. Out of 100 cases, 89% of cases were normal or underweight; and 9% and 2% of the cases were overweight and obese, respectively (Table 8).

**Table 8:** Distribution of study subjects based on BMI BMI: body mass index.

BMI	Frequency	Percentage
Normal/underweight (<25)	89	89
Overweight (25-30)	9	9
Obese (>30)	2	2

Upper GI endoscopy

All the patients taken in our study underwent upper GI endoscopy. Out of 100 cases, 55 cases had normal study in their upper GI endoscopy, while 45 cases had evidence of lesions suggestive of GERD (Table 9). Among these cases, Grades A, B, C, and D lesions were found in 21, 18, four, and two cases, respectively (Table 10).

**Table 9:** Distribution of patients based on upper GI endoscopic findings NERD: non-erosive reflux disease, ERD: erosive reflux disease.

Endoscopy Report	Frequency	Percentage
Lesion absent (NERD)	55	55
Lesion present (ERD)	45	45
Grade A	21	21
Grade B	18	18
Grade C	4	4
Grade D	2	2
Total	100	100

**Table 10:** Distribution of patients with lesions based on LA Classification GERD: Gastroesophageal reflux disease, LA: Los Angeles.

Grading of GERD	Frequency	Percentage
Grade A	21	46.7
Grade B	18	40
Grade C	4	8.8
Grade D	2	4.5
Total	45	100

Correlation between symptomatology and endoscopic findings in GERD

Correlation Between the Symptoms of GERD With Its Endoscopic Findings

Each symptom of GERD is correlated with their upper GI endoscopy findings. Among 100 cases, typical symptoms like heartburn, regurgitation, and epigastric pain were present in 63, 71, and 78 cases, respectively, out of which erosive lesions were present in 24, 37, and 38 cases, respectively. Using the Chi-Square test, these typical symptoms with their endoscopic findings were compared and the p-value was calculated. A significant p-value was found in regurgitation symptoms only, while others had an insignificant p-value (Table 11).

**Table 11:** Correlation between typical symptoms of GERD and its endoscopic findings GERD: gastroesophageal reflux disease, NERD: non-erosive reflux disease, ERD: erosive reflux disease.

Typical Symptoms	Lesions Absent (NERD)	Lesions Present (ERD)	X^2 ^Value	p-Value
Heartburn	39	24	3.279	0.07
Regurgitation	34	37	5.004	0.02
Epigastric pain	40	38	1.980	0.15

Similarly, among 100 cases, atypical symptoms like dysphagia, hoarseness, sore throat, bloating, belching, and vomiting were present in nine, eight, five, eight, 16, and seven cases, respectively. Like typical symptoms, these atypical symptoms were also compared with their endoscopic findings using the Chi-Square test. No significant p-value was found in any of the atypical symptoms correlation (Table 12).

**Table 12:** Correlation between atypical symptoms of GERD and its endoscopic findings GERD: gastroesophageal reflux disease, NERD: non-erosive reflux disease, ERD: erosive reflux disease.

Atypical Symptoms	Lesions Absent (NERD)	Lesions Present (ERD)	*X*^2^ Value	p-Value
Dysphagia	5	4	0.001	0.97
Hoarseness	4	4	0.087	0.76
Sore throat	4	1	1.329	0.24
Bloating	6	2	1.405	0.23
Belching	8	8	0.192	0.66
Vomiting	4	3	0.013	0.90

Correlation Between the Duration of Symptoms With Its Endoscopic Findings

Based on the duration of symptoms, cases were categorically classified into three groups, i.e., less than four months, four to six months, and more than six months. Thirty-two cases had less than four months duration, out of which 12 cases had erosive lesions in their endoscopy. Similarly, 52 and 16 cases were having symptoms for four to six months and more than six months, respectively, out of which 24 and nine cases had erosive lesions, respectively. Using the Chi-Square test, the duration of symptoms was compared with their endoscopic findings and p-value was calculated and found to be 0.45 which is statistically insignificant (Table 13).

**Table 13:** Correlation between duration of symptoms and its endoscopic findings NERD: non-erosive reflux disease, ERD: erosive reflux disease.

Duration	Lesions Absent (NERD)	Lesions Present (ERD)
Less than four months	20	12
Four to six months	28	24
More than six months	7	9
Total	55	45
X^2^ value	1.573	
p-value	0.45	

Correlation Between the Frequency of Symptoms With Its Endoscopic Findings

Based on the frequency of symptoms, cases were categorically classified into four groups, i.e., once in a month, once in a week, several times in a week, and daily. Seventeen cases had symptoms once in a month, out of which eight cases had erosive lesions in their endoscopy. Similarly, 24, 28, and 31 cases had symptom frequency of once in a week, several times in a week, and daily, respectively, out of which 16, 15, and 15 cases had erosive lesions, respectively. Using the Chi-Square test, the frequency of symptoms was compared with their endoscopic findings and p-value was calculated and found to be 0.58 which is statistically insignificant (Table 14).

**Table 14:** Correlation between frequency of symptoms and its endoscopic findings NERD: non-erosive reflux disease, ERD: erosive reflux disease.

Frequency	Lesions Absent (NERD)	Lesions Present (ERD)
Once in a month	9	8
Once in a week	16	8
Several times in a week	15	13
Daily	15	16
Total	55	45
*X*^2^ value	1.919	
p-value	0.58	

Correlation Between the Severity of Symptoms With Its Endoscopic Findings

Based on the severity of symptoms, cases were categorically classified into three groups, i.e., mild, moderate, and severe. Fifty-eight cases had mild symptoms, out of which 25 cases had erosive lesions in their endoscopy. Similarly, 24 and 18 cases had moderate and severe symptoms, respectively, out of which 14 and eight cases had erosive lesions, respectively. Using the Chi-Square test, the frequency of symptoms was compared with their endoscopic findings and the p-value was calculated and found to be 0.60 which is statistically insignificant (Table 15).

**Table 15:** Correlation between severity of symptoms and its endoscopic findings NERD: non-erosive reflux disease, ERD: erosive reflux disease.

Severity	Lesions Absent (NERD)	Lesions Present (ERD)
Mild	33	25
Moderate	14	10
Severe	8	10
Total	55	45
*X*^2^ value	1.002	
p-value	0.60	

Contributing factors for the development of erosive lesions in GERD

Various contributing factors for the development of erosive lesions in GERD in our study are thought to be a sedentary lifestyle, insomnia, overweight/obesity, and addictive history of alcohol consumption, smoking, and tobacco consumption. They were found in 10, eight, 13, 21, 15, and 14 cases, respectively, out of which seven, seven, 10, 11, six, and six cases had erosive lesions, respectively, in their endoscopy. Using the Chi-Square test, each of the contributing factors was compared with their endoscopic findings and the p-value was calculated. Insomnia and overweight/obesity factors had a p-value of 0.01 and 0.014 which is considered statistically significant. The rest of the factors were statistically insignificant (Table 16).

**Table 16:** Contributing factors for the development of erosive lesions in GERD GERD: gastroesophageal reflux disease, NERD: non-erosive reflux disease, ERD: erosive reflux disease.

Contributing Factors	Lesions Absent (NERD)	Lesions Present (ERD)	*X*^2^ Value	p-Value
Sedentary lifestyle	3	7	2.805	0.09
Insomnia	1	7	3.711	0.01
Alcohol	10	11	0.585	0.44
Smoking	9	6	0.178	0.67
Tobacco	8	6	0.030	0.86
Overweight/obese	3	10	5.923	0.014

## Discussion

Discomfort in the upper abdomen is one of the most common presenting complaints. Gastroesophageal reflux disease (GERD) is one such disease that causes it frequently and is also the usual culprit damaging the esophagus. There are no specific diagnostic criteria that are convenient and easily available that can detect all the cases of GERD. Henceforth, it is less detected and treated only when a patient has landed up in complications due to it. However, it has not stopped fascinating the researchers as well as clinicians with its wide range of presentation, epidemiological change, unavailability of gold-standard tests to diagnose it, and emerging treatment. In the Western world, the major population getting affected by GERD is the adult age group. Several studies had found that the occurrence of it in Asia is increasing day by day. The reason behind this could be increased transformation of our lifestyle to the Western world, improved clinical diagnosis by skilled registered medical practitioners, and increased availability of advanced diagnostic modalities at a feasible value [13]. Even after it is diagnosed with increased numbers at an early phase in today’s world, pathophysiology, as well as etiology, is still unclear or imperfect [14]. Hence, GERD is defined on the basis of three important criteria which are a doctor’s clinical assessment, evaluation of the esophagus for erosions by upper gastrointestinal endoscopy, and ambulatory 24-hour pH monitoring of the esophagus [15].

This particular clinical research has been carried out to study the clinical spectrum of GERD, endoscopic evaluation of GERD, the contribution of risk factors, and the correlation of clinical symptoms with the endoscopic findings. Many of the findings and results of this study have been well correlated with such clinical research done earlier. In our study, out of 100 cases, 58 cases were male with a male:female ratio of 1.38:1 suggesting mild male preponderance. However, the p-value was insignificant suggestive of equivocal distribution among both genders. Similar studies done by Tidake et al. showed a ratio of 1.08:1 [16]. Zuberi et al. showed a ratio of 0.97:1 [13]. Du et al. showed a ratio of 1.30:1 and Nocon et al. showed a male:female ratio of 1.12:1 [13,15-17] (Table 17). Most of the patients taken in the study were in the age group of 30-60 years constituting 70% of total cases with a mean age of 44.82 years. Even various similar studies like Tidake et al. and Wang et al. showed an incidence of 50% and 70%, respectively, among the same age group of 30-60 years [16,18]. Similarly, a study by Albayati et al. showed an incidence of 42% of esophageal erosions [19].

**Table 17:** Percentage of patients with erosive lesions on endoscopic evaluation in various similar studies SRTR: Swami Ramanand Teerth Rural Medical College.

S.No	Research Study	Percentage
1	Zuberi et al., Dow University of Health Sciences, Karachi, Pakistan [13]	44.4
2	Vaishnav et al., Dr. D. Y. Patil Medical College, Pune, India [14]	51.7
3	Meira et al., Universidade Estadual do Sudoeste da Bahia, BA, Brasil [20]	42
4	Du et al., College of Medicine, Zhejiang University, China [15]	20.8
5	Tidake et al., SRTR Ambajogai, Beed, India [16]	48
6	Albayati and Khalaf, College of Medicine, Mustansiriyah University, Baghdad, Iraq [19]	42
7	Our study	45

Typical symptoms comprised heartburn, regurgitation, and epigastric pain. Most of the cases had epigastric pain constituting 78% followed by regurgitation and heartburn comprising 71% and 63%, respectively. A study done by Tidake et al. also showed similar values of heartburn, regurgitation, and epigastric pain with 94%, 80%, and 22%, respectively [16]. The present study was done with special attention to endoscopic evaluation. Thus, upper GI endoscopy was done in all cases taken in the study. Symptoms of GERD with the normal endoscopic study were categorized as non-erosive reflux disease (NERD), while its counterpart is named erosive reflux disease (ERD). Fifty-five percent of the cases had a normal study, while the other had erosive lesions in the esophagus which were graded as per Los Angeles (LA) classification. Relative studies also revealed a mild dominance of NERD over ERD. Henceforth, there is a limitation of the low sensitivity of endoscopic evaluation for GERD. Thus, ambulatory 24-hour pH monitoring is considered the gold standard to diagnose GERD. However, it also has its problems with sensitivity due to intermittent symptomatology and disturbance caused by the pH probe placement following daily activities [15]. This is the reason why correlating symptoms along with endoscopic findings plays a vital role in making decisions regarding diagnosis as well as treatment.

Each of the typical symptoms as well as the atypical symptoms was correlated with their endoscopic findings. Surprisingly, no significant correlation was established in all the cases due to their equitable distribution in both NERD and ERD except for regurgitation symptoms which were found statistically significant (p-value=0.02) since 37 cases out of 71 with regurgitation symptoms had erosive lesions. Duration of symptoms, frequency of symptoms, and severity of symptoms were also correlated with the endoscopic findings. Most of the cases had symptoms for four to six months (52%), mild severity (58%), and frequency of symptoms daily (31%). However, there was no statistically significant correlation established between them and endoscopic evaluation.

There is evidence of an increased incidence of GERD in India. A sedentary lifestyle could be an attributable risk factor for this. In our study, 10% of the patients had a sedentary lifestyle out of which seven cases had erosive lesions on their endoscopic evaluation, while three cases had normal endoscopic study.

Insomnia is a symptom of a sleep disorder, wherein the patient has difficulty in maintaining or initiating sleep. Jung et al. have done a study that revealed that there is a bidirectional association between GERD and insomnia [20]. In our study, among 100 cases, eight patients had a history of insomnia out of which seven cases had erosive lesions suggesting a statistically significant correlation between insomnia and GERD.

Alcohol consumption, smoking, and tobacco chewing are considered risk factors for the development of GERD symptoms. Thus, addictive history was assessed in all the cases taken in our study. It was found that 11 (24.4%) cases out of 45 cases having erosive lesions on endoscopic evaluation had a history of alcohol consumption, whereas 10 (18.2%) cases out of 55 cases having a normal study on endoscopic evaluation had a history of alcohol consumption. In a similar study done by Meira et al. and Labenz et al., 36.84% and 70% among the cases with erosive lesions and 33.25% and 63% among the cases with non-erosive lesions had a history of alcohol consumption, respectively [9,21]. Among all 45 cases with erosive lesions on endoscopic evaluation, six (13.3%) cases had smoking addiction, while nine (16.3%) cases among 55 cases with the normal endoscopic study had smoking addiction. In a similar study done by Meira et al., Labenz et al., and Albayati and Khalaf, 7.32%, 58%, and 54.7% among erosive lesions and 5.12%, 48%, and 8.6% among non-erosive lesions had smoking addiction, respectively [9,19,21]. Tobacco was seen in 13.3% of cases with erosive lesions, while tobacco was seen in 14.5% among cases with non-erosive lesions. However, no statistically significant correlation could be established between addictive history and GERD, possibly due to subjective errors as many do not reveal their addictions during history-taking.

One of the independent risk factors for GERD and hiatus hernia is obesity or excessive body weight [14]. However, there is an uncertainty in the relation between GERD and obesity. But a study by Nilsson et al. demonstrates a strong association between increasing body mass index and symptomatic reflux in women and a moderate association among men [22]. In our study, among 100 cases, 13 patients had a BMI of >25. In a study done by Wang et al., 33.33% of cases had a BMI of >25 [18]. Out of 45 cases having erosive lesions on endoscopy, 10 (22.2%) cases were overweight/obese, while out of 55 cases having non-erosive lesions on endoscopy, three (5.4%) cases were overweight/obese. In a similar study done by Vaishnav et al., 46% among erosive lesions and 28.2% among non-erosive lesions were overweight/obese [14]. It was found that there was a statistically significant correlation between obesity and GERD (p-value=0.014) in our study.

## Conclusions

This was a descriptive study done among the patients visiting the surgery, medicine, or gastroenterology OPD clinic at AVBRH, affiliated to JNMC, Sawangi (Meghe), Wardha, between September 2019 to December 2021, with symptoms suggestive of gastroesophageal reflux disease (GERD). A detailed clinical history including demographic details, duration, frequency and severity of symptoms, and possible risk factors like sedentary lifestyle, insomnia, smoking/alcohol/tobacco, height, weight, and other necessary information was taken and entered in a proforma. The patient was then convinced to undergo upper GI endoscopy, findings of which were recorded appropriately. Reflux esophagitis noted on endoscopic evaluation was graded as per LA classification. Using the obtained data, clinical spectrum, endoscopic evaluation, the correlation between symptoms and endoscopic evaluation, and contribution of risk factors in developing esophageal erosions were studied. A total of 100 patients were included in the study. A number of male patients (58%) were more than that of female patients. Most of the patients were in the age group of 30-60 years (70%). The most common symptoms were epigastric pain (78%), regurgitation (71%), and heartburn (63%). Atypical symptoms included dysphagia, hoarseness, sore throat, bloating, belching, and vomiting. A sedentary lifestyle was present in 10%. Insomnia was present in 8%. History of alcohol consumption, smoking, and tobacco chewing was present in 21%, 15%, and 14%, respectively. Overweight/obese patients as per BMI (13% of the cases) were present. Forty-five percent of the patients had erosive lesions suggestive of reflux esophagitis on endoscopic evaluation. LA Grade A (46.7%) was the most common finding among patients with erosive lesions. Among the symptoms, only regurgitation (p=0.02) showed a significant correlation with endoscopic findings.

Among contributing factors, insomnia (p=0.01) and obesity (0.014) showed positive correlation with esophageal lesions. Incidence of GERD symptoms is more common in the age group of 30-60 years with mild male preponderance. The normal endoscopic finding is not uncommon among patients with GERD symptoms. There is a poor association between the duration, frequency, and severity of symptoms of GERD and the presence of erosive lesions. Among symptoms, only regurgitation has a statistically significant correlation. Among contributing risk factors, only insomnia and obesity have a statistically significant correlation.

All patients with symptoms of GERD may not be associated with evidence of erosive lesions suggestive of reflux esophagitis on endoscopic evaluation. This reinforces the fact that non-erosive reflux disease (NERD) is a common entity, which forms a significant proportion of patients seeking medical attention for reflux symptoms. However, since the patients with erosive lesions, Barrett’s esophagus, are at a higher risk of developing esophageal adenocarcinoma in the future, it is suggested that all the patients with symptomatic GERD should be subjected to a simple, easily feasible, and least harmful endoscopic evaluation and biopsy if needed.
